# Vasopressin and oxytocin in sensory neurones: expression, exocytotic release and regulation by lactation

**DOI:** 10.1038/s41598-018-31361-1

**Published:** 2018-08-30

**Authors:** Govindan Dayanithi, Oksana Forostyak, Serhiy Forostyak, Tomohiko Kayano, Yoichi Ueta, Alexei Verkhratsky

**Affiliations:** 1 Institut des Sciences Biologiques-Neurosciences, cognition, Centre Nationale de la Recherche Scientifique, 3 rue Michel-Ange, 75794 Paris cedex 16, France; 2 MMDN-Institut National de la Santé et de la Recherche Médicale-U1198, Université de Montpellier, 34095 Montpellier, France; 3 Ecole Pratique des Hautes Etudes, Sorbonne, Les Patios Saint-Jaques, 75014 Paris, France; 4 Department of Pharmacology and Toxicology, Faculty of Medicine, Charles University at Plzen, CZ-32300 Plzen, Czech Republic; 5 Department of Molecular Neurophysiology, Institute of Experimental Medicine, Czech Academy of Sciences, 14220 Prague, Czech Republic; 6 Department of Neuroscience, 2nd faculty of Medicine, Charles University, V Uvalu 84, 15006 Prague, Czech Republic; 7 PrimeCell Therapeutics a.s. Palachovo Náměstí 2, 625 00 Brno, Czech Republic; 8 Department of Physiology, School of Medicine, University of Occupational and Environmental Health, Kitakyushu, 807-8555 Japan; 9 Faculty of Biology, Medicine and Health, University of Manchester, M13 9PT Manchester, UK; 10 Achucarro Centre for Neuroscience, IKERBASQUE, Basque Foundation for Science, 48011 Bilbao, Spain

## Abstract

The neurohormones arginine-vasopressin (AVP) and oxytocin (OT) synthesised in supraoptic and paraventricular nuclei of neurohypophysis regulate lactation, systemic water homeostasis and nociception. Using transgenic rats expressing AVP and OT tagged with fluorescent proteins we demonstrate that both neurohormones are expressed in sensory neurones both *in vitro*, in primary cultures, and *in situ*, in the intact ganglia; this expression was further confirmed with immunocytochemistry. Both neurohormones were expressed in nociceptive neurones immunopositive to transient receptor potential vannilloid 1 (TRPV1) channel antibodies. The AVP and OT-expressing DRG neurones responded to AVP, OT, 50 mM K^+^ and capsaicin with [Ca^2+^]_i_ transients; responses to AVP and OT were specifically blocked by the antagonists of V_1_ AVP and OT receptors. Probing the extracellular incubation saline with ELISA revealed AVP and OT secretion from isolated DRGs; this secretion was inhibited by tetanus toxin (TeNT) indicating the role for vesicular release. Expression of OT, but not AVP in DRG neurones significantly increased during lactation. Together, the results indicate novel physiological roles (possibly related to nociception and mood regulation) of AVP and OT in the sensory neurones.

## Introduction

It is a truth universally acknowledged that neurohypophyseal hormones arginine vasopressin (AVP) and oxytocin (OT) are synthesized in the magnocellular neurosecertory cells (MNCs) of the paraventricular and the supraoptic nuclei (PVN and SON respectively) of the hypothalamus. These hormones are secreted from MNCs axons that terminate in the posterior pituitary into the systemic circulation; OT and AVP secretion is linked to highly idiosyncratic electrical activities of MNCs^1^. The neurohormones exert multiple effects on peripheral tissues and cells through activating dedicated metabotropic receptors for vasopressin (V_1a_, V_1b_ and V_2_) and oxytocin (OT-R) with well defined pharmacology^2–5^.

Several sporadic studies reported expression of AVP and OT outside of the neurohypophysis; immunoreactivity for both hormones was, for example, detected in ~50% of rat dorsal root ganglia (DRG) neurones^6,7^. Similarly, oxytocin was found (by radioimmunoassay) in human lumbar DRGs^8^. These observations, however, were not universally confirmed; neither AVP nor OT were identified in DRGs from guinea-pig, cat, rat or rabbit^9–11^. Exposure of DRG neurones to AVP or OT caused accumulation of inositol phosphates, suggesting the presence of functional metabotropic (V_1_) receptors^12^. Oxytocin is known to exert analgesic properties and strong spinal anti-nociceptive action^13,14^. Analgesia induced by systemic applications of OT is mediated by V_1a_ receptors expressed in DRG neurones^15,16^. Both AVP and OT modulate nociception and pain responses by direct activation of AVP-V_1a_ and OT receptors^17,18^.

A novel methodology based on the expression of AVP or OT fused with fluorescent proteins (eGFP, eCFP or mRFP1) in rats allowed further insights into the neurobiology of these hormones^19^. These transgenic rats are valuable tools to identify AVP and OT-expressing neurones and their terminals they can also be used for monitoring dynamic changes in AVP and OT expression in physiological and pathological contexts^5,20–23^. Using these transgenic rat models we, for the first time, unequivocally visualized AVP and OT in live sensory neurones. Both neurohormones are expressed in nociceptive sensory neurones as judged by functional expression of TRPV1 channels. Probing media in which DRGs were incubated with ELISA essay revealed secretion of AVP and OT that was inhibited by tetanus toxin (TeNT) indicating the role for exocytosis. Expression of OT in DRG neurones was substantially up-regulated in lactation. Preliminary results appeared as abstract form^24,25^.

## Results

### Expression of AVP and OT in DRG neurones *in situ* and *in vitr****o***

**Figure 1:**
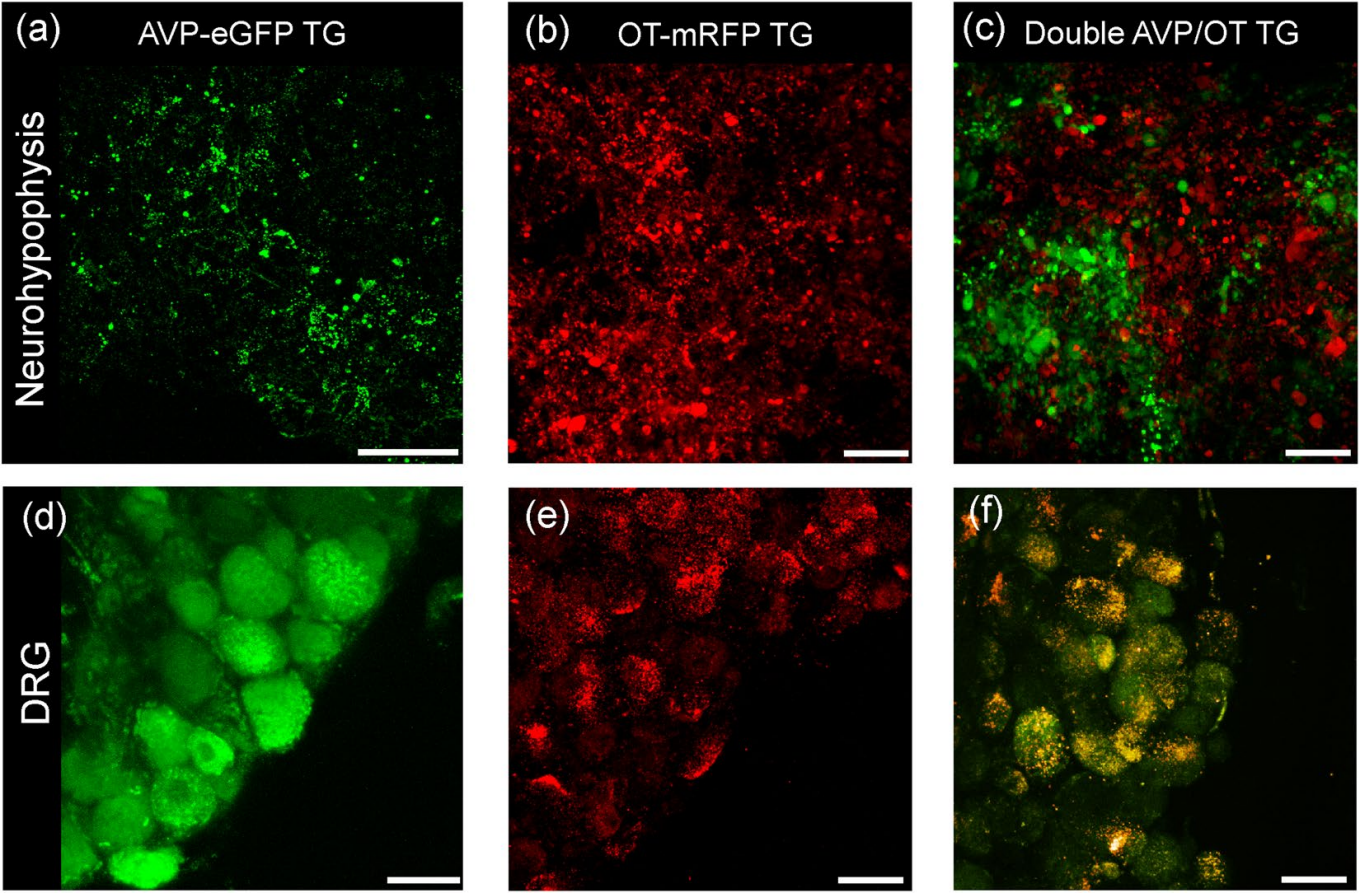
Expression and visualization of fluorescent AVP and OT in neurohypophysis and in DRGs in transgenic rats. Confocal images showing AVP and OT fluorescence associated with neuronal cell bodies in freshly isolated neurohypophysis (NH), (**a**–**c**) and in DRG preparations (**d**–**f**) from transgenic AVP-eGFP (**a**,**d**), OT-mRFP (**b**,**e**) and AVP-eGFP/OT-mRFP transgenic homozygote male rats (**c**,**f**). Note clear separation of AVP and OT- fluorescent neurones in MH vs. co-localisation of both fluorescent signals in DRG neurones. Scale bars 50 μm.

The whole DRGs from the 6–8 weeks old AVP-eGFP, OT-eCFP, OT-mRFP or AVP-eGFP/OT-mRFP double transgenic male rats^19,21,26,27^ were freshly isolated and analysed under confocal microscope. To reassure proper expression of fluorescent protein tagged neurohormones the neurohypophyses were isolated from each animal and the tissues were examined under confocal microscope to confirm neurohypophyseal expression of AVP- or OT-related fluorescence (Fig. 1a–c). In AVP-eGFP rats the fluorescence could be detected in both middle size and large DRG neurones (Fig. 1d). Experiments on OT-eCFP transgenic rat model similarly revealed eCFP fluorescence in the DRG neurones, however several technical issues such as instability of the fluorescence expression, rapid photo bleaching and breeding problems made the use of this strain somewhat problematic. Therefore the experiments were repeated using OT-mRFP transgenic rat model that was stable and devoid of complications outlined above. The expression of endogenous mRFP fluorescence in OT-mRFP transgenic rats confirmed the presence of OT in DRG neurones (Fig. 1e). In the double transgenic rats expressing tagged AVP and OT, the neurohypophysis showed clear separation of two neuronal (AVP and OT) populations (Fig. 1c), whereas in DRG preparations we observed co-localisation of both hormones within same neurones (Fig. 1f).

**Figure 2:**
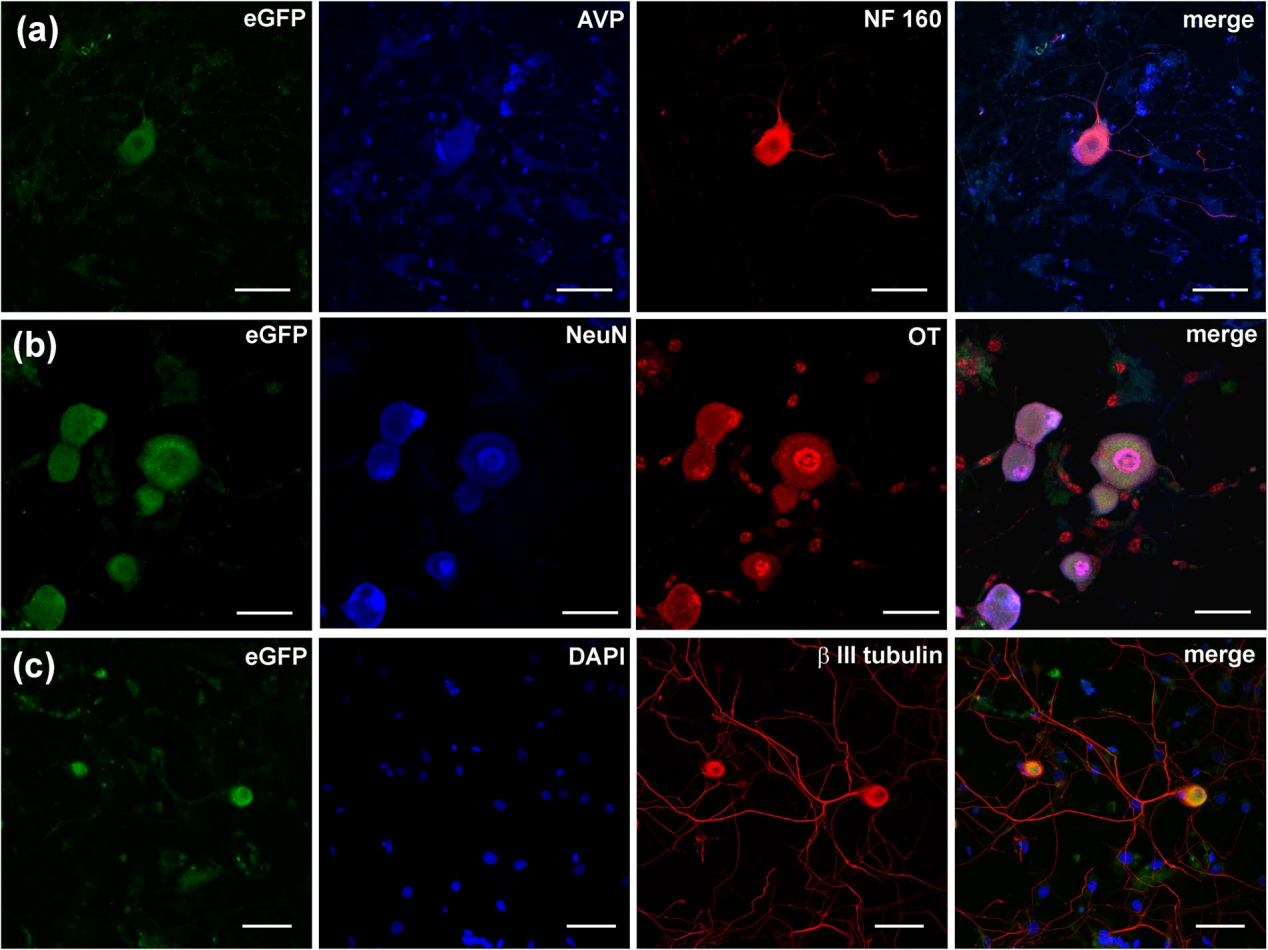
Immunohystochemical labelling of AVP-eGFP fluorescent DRG neurones in culture. DRG neurones expressing AVP-eGFP were stained with antibodies against AVP and NF160 (**a**), NeuN and OT (**b**) and DAPI and βIII tubulin (**c**). Endogenous AVP-eGFP fluorescence in NeuN (+) cells is co-localized with the staining against OT (**b**), suggesting that neuronal NeuN (+) cells express both AVP and OT. Scale bars 50 μm.

**Figure 3:**
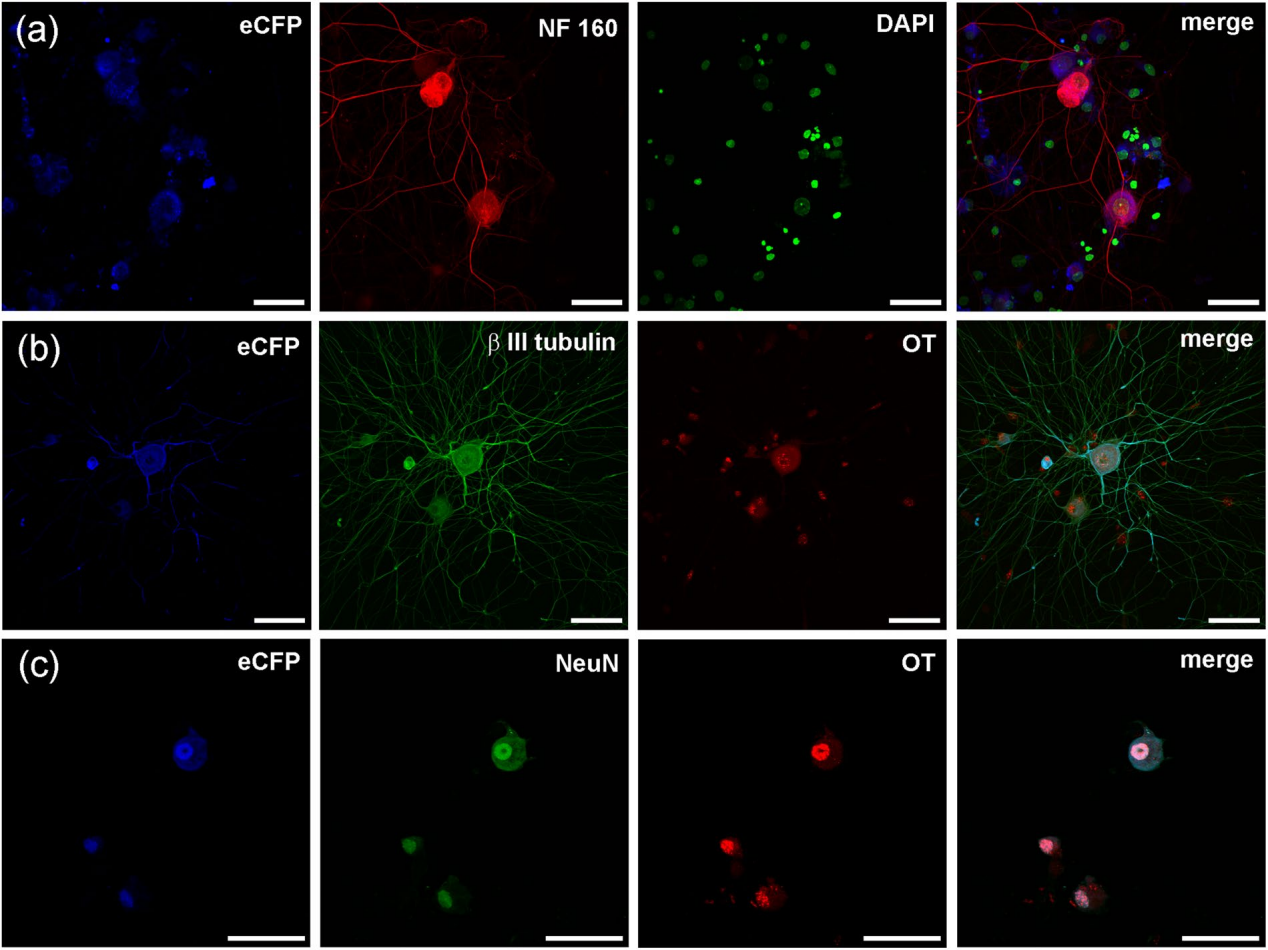
Immunohystochemical labelling of OT-cCFP fluorescent DRG neurones in culture. DRG neurones expressing OT-eCFP stained with antibodies against NF160 and DAPI (**a**), βIII tubulin and OT (**b**) and NeuN and OT and (**c**). Scale bars 50 μm.

**Figure 4:**
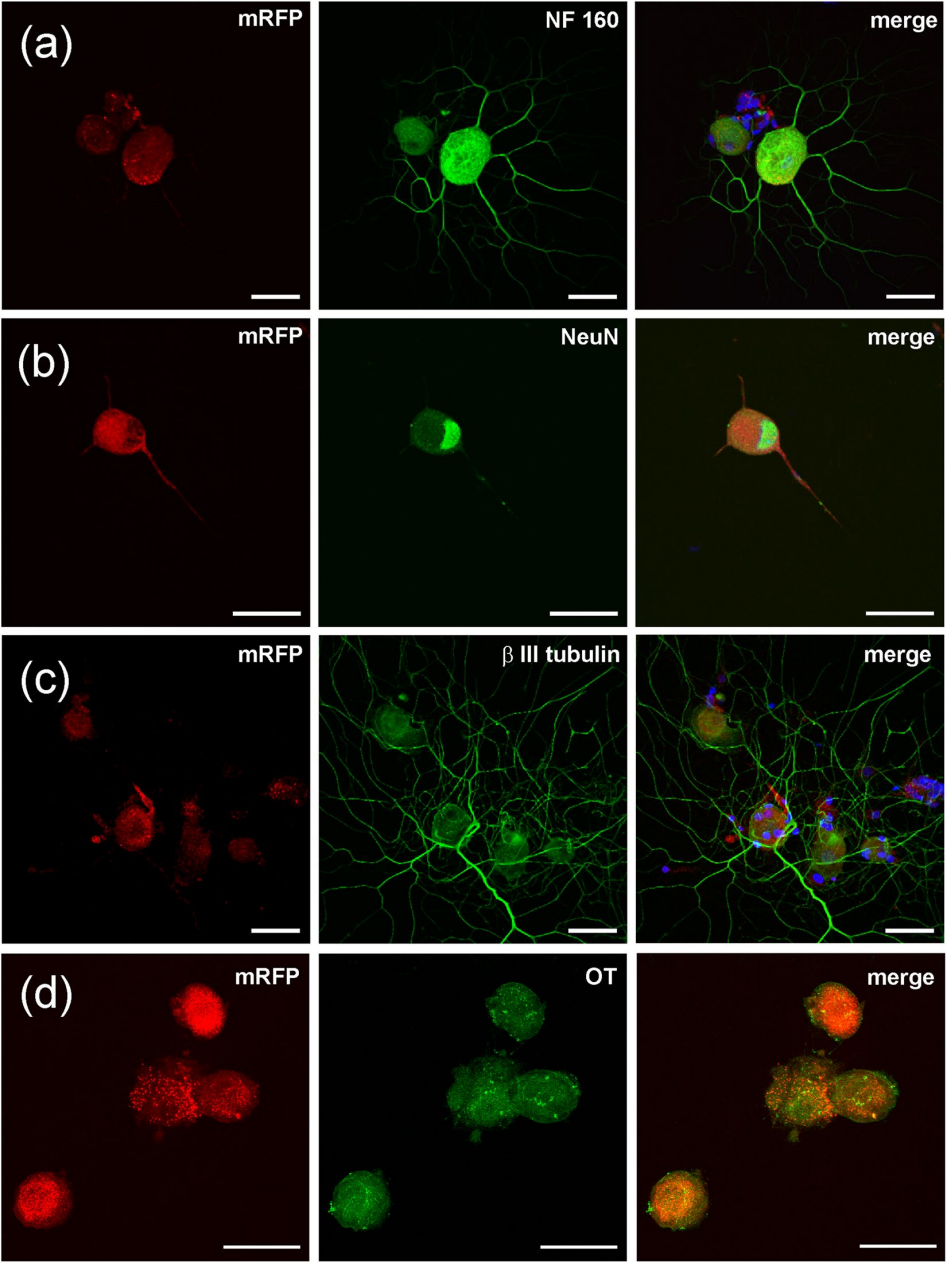
Immunohystochemical labelling of OT-mRFP fluorescent DRG neurones in culture. DRG neurones expressing OT-mRFP were stained with antibodies against NF160 (**a**), NeuN (**b**) or βIII tubulin (**b**) and Scale bars 50 μm.

Cells expressing AVP and OT were further characterised by an *in vitro* immunocytochemistry. Cultured (48 hours) DRG cells isolated from AVP-eGFP transgenic rats were stained with the antibodies against AVP, βIII tubulin, NeuN, NF160, and OT. Cells expressing AVP-linked eGFP marker were immunopositive for AVP antibody (Fig. 2a) as well as for neuronal markers NF160 (Fig. 2a), NeuN (Fig. 2b) and βIII tubulin (Fig. 2c). The AVP-eGFP fluorescent cells also demonstrated immunoreactivity for OT (Fig. 2b), which further corroborated simultaneous expression of both hormones in the same DRG neurone. Cell cultures prepared from DRGs isolated from OT-eCFP (Fig. 3) and OT-mRFP (Fig. 4) rats demonstrated co-localisation of OT fluorescence with immunopositivity to OT and neuronal markers NF160, NeuN and βIII tubulin.

### Vasopressin and oxytocin are expressed in nociceptive neurones

**Figure 5:**
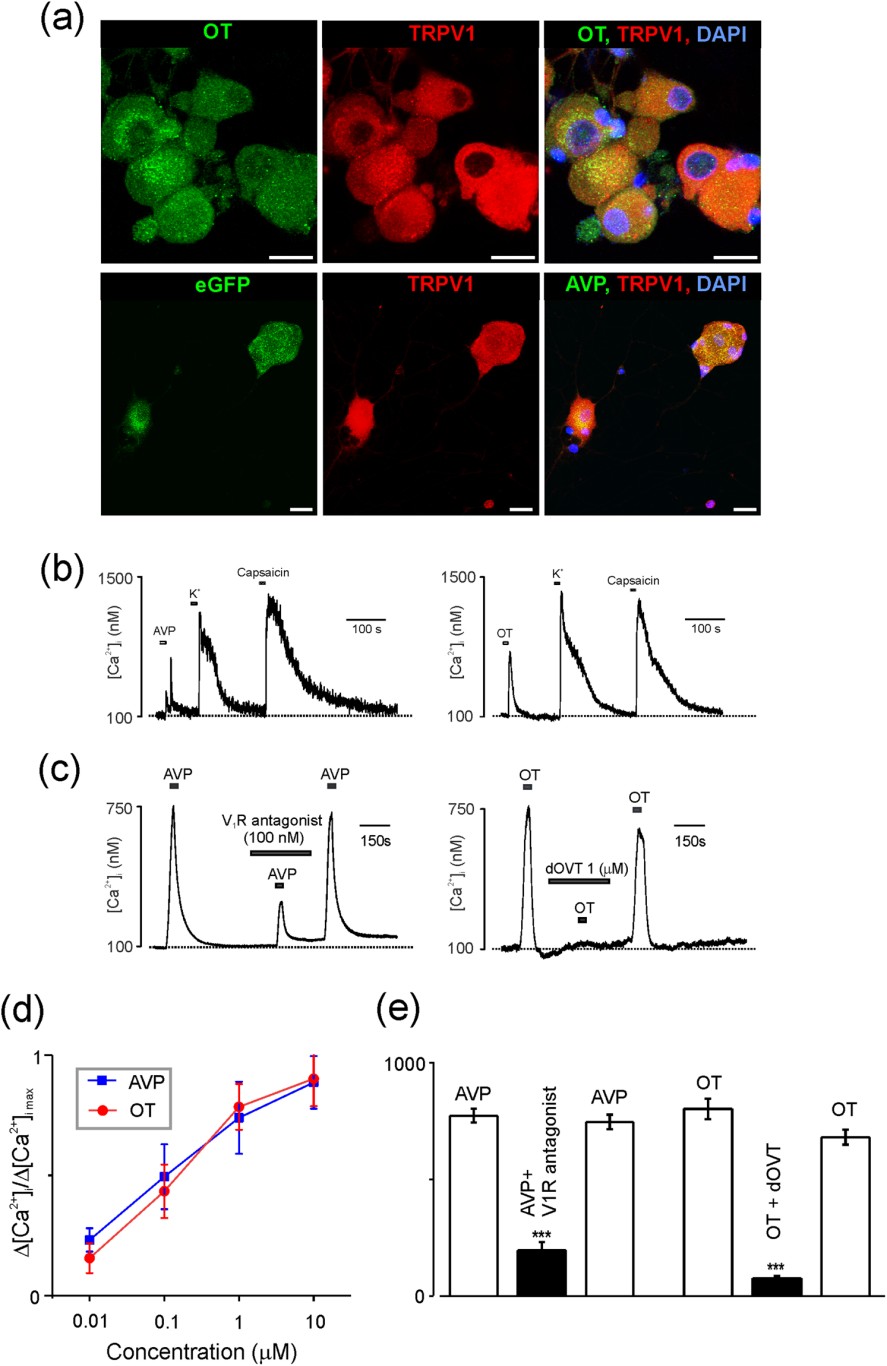
OT and AVP are expressed in nociceptive DRG neurones. (**a**) Top panel: Confocal images DRG neurones cultured for 48 hours stained with antibodies against OT and TRPV1; the merged image shows co-localisation of both markers. Bottom panel: Confocal images of AVP-eGFP DRG neurones cultured for 48 hours and stained with TRPV1 antibody. The merged image shows co-localisation of both markers. Scale bars 50 μm. (**b**) AVP, OT and capsaicin-induced [Ca^2+^]_i_ responses in cultured DRG neurones. Representative traces showing [Ca^2+^]_i_ responses to AVP (100 nM), OT (100 nM), K^+^ (50 mM) and capsaicin (1 μM). (**c**) Pharmacology of AVP and OT-induced [Ca^2+^]_i_ responses. Traces show AVP or OT-induced [Ca^2+^]_i_ transients in control, in the presence of specific antagonist ([deamino-Pen^1^, O-Me-Tyr^2^, Arg^8^]-vasopressin or dOVT) and after washout. (**d**) Concentration dependence of AVP and OT-induced Ca^2+^ signals. The graph shows average peak amplitudes of [Ca^2+^]_i_ transients at different concentrations of the agonist. (amplitudes are presented as mean ± SEM; experiments were performed on 6 AVP-eGFP neurones from 3 different cultures and 6 OT-mRFP neurones from 3 different cultures). (**e**) Average amplitudes (mean ± SEM), of [Ca^2+^]_i_ transients triggered by AVP and OT in control conditions and in the presence of specific inhibitors of V_1a_ receptors ([deamino-Pen^1^, O-Me-Tyr^2^, Arg^8^]-vasopressin; n = 6 AVP-eGFP neurones from 3 cultures; p = 0.0094, Friedman test) or OT receptors (dOVT; n = 5 OT-mRFP neurones from 3 cultures p = 0.0067, Friedman test).

Dorsal root ganglia contain cell bodies of the first order neurones of the ascending somatosensory pathways. These neurones receive proprioceptive, mechanosensory, thermoreceptive or nociceptive information from muscles, tendons and skin^28^. Various types of sensory transduction are provided by the members of transient receptor potential (TRP) family^29^; with a particular role for vanilloid TRP channels (TRPV). Immunostaining of cell cultures prepared from DRGs isolated from three AVP-eGFP or three OT-mRFP rats with antibodies against TRPV1 channel revealed co-localisation of AVP as well as OT related fluorescence with expression of TRPV1 channels (Fig. 5a). The AVP was expressed in 37 out of 44 TRPV1-positive neurones, whereas OT was found in 144 out of 148 TRPV1-positive cells (images were taken from three different cultures). Expression of TRPV1, AVP and OT was detected only in neurones; cells with fibroblast or glial morphology expressed neither hormones nor TRPV1 channels.

### Nociceptive neurones express functional AVP and OT receptors

Functional activity of AVP and OT neurones was monitored by recording cytosolic Ca^2+^ concentration ([Ca^2+^]_i_) by video-imaging or by single-cell photometry. All cells were probed with high-K^+^ depolarisation to confirm their excitability and hence neuronal identity. Out of 112 AVP-eGFP neurones 80 responded with transient [Ca^2+^]_i_ elevation to application of AVP, while OT triggered [Ca^2+^]_i_ transients in 33 out of 44 OT-mRFP neurones. Finally 14 out of 14 “double transgenic” AVP-eGFP/OT-mRFP neurones responded with [Ca^2+^]_i_ transients to sequential application of both AVP and OT.

Both AVP and OT triggered transient [Ca^2+^]_i_ increase in dose-dependent manner (Fig. 5b,d) with EC_50_ of 431 nM and 417 nM, respectively. All cells sensitive to AVP or OT also generated [Ca^2+^]_i_ transients in response to depolarisation with 50 mM KCl and to administration of TRPV1 agonist 1 μM capsaicin (Fig. 5b). Responses to capsaicin were reversibly blocked by specific TRPV1 antagonist capsazepine (10 μM; n = 5, data not shown). These data together indicate that receptors for AVP and OT are expressed in nociceptive neurones. [Ca^2+^]_i_ transients triggered by neurohormones were sensitive to specific antagonists of V_1_ and OT receptors (Fig. 5c,e). Incubation of neurones with 1 μM of V_1_ antagonist ([deamino-Pen^1^, O-Me-Tyr^2^, Arg^8^]-vasopressin) decreased the amplitude of 100 nM AVP-induced [Ca^2+^]_i_ transients from 773 ± 30 nM to 196 ± 35 nM, n = 6, p ≤ 0.001). Similarly, incubation with 1 μM of OTR antagonist dOVT decreased the amplitude of OT (100 nM)-evoked [Ca^2+^]_i_ transient from 790 ± 43 nM, to 71 ± 11 nM, n = 5; p ≤ 0.001.

### DRG neurones release of AVP and OT is sensitive to tetanus toxin

**Figure 6:**
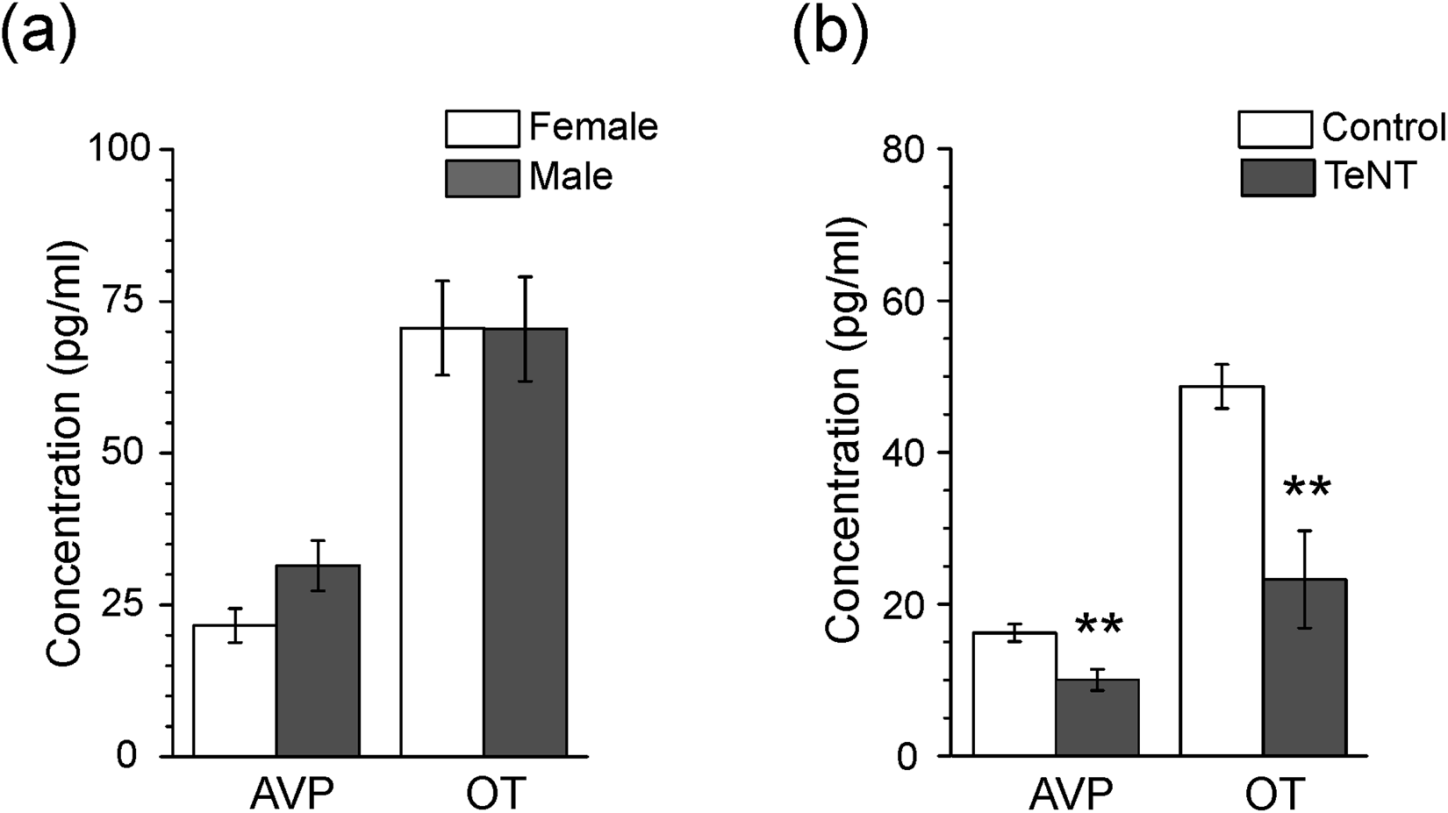
AVP and OT release form DRG neurones is inhibited be tetanus toxin. Concentration of AVP and OT in the media containing DRGs was measured with ELISA assay. Each sample contained 10 ganglia isolated from different levels of spinal cord. (**a**) A bar graph shows the content of AVP and OT in the media containing DRG isolated from female and male rats. (**b**) The overnight incubation of DRGs (isolated from male rats) with 100 nM of tetanus toxin caused a significant decrease in AVP and OT content in the media (AVP: p = 0.0037; OT: p = 0.0036, two-sample t-test).

The release of AVP and OT from DRG neurones was analysed with competitive ELISA. For each experiment, 10 DRG ganglia isolated from different regions of spinal cord of 4 weeks old female and male Wistar rats were used (see methods for details). The AVP and OT concentration was determined in the incubation media after overnight incubation. Similar levels of OT and AVP have been detected in control males and females groups (Fig. 6a). The concentration of AVP in the incubation media containing DRGs isolated from female and male rats was 21.61 ± 2.8 pg/ml and 31.44 ± 4.1 pg/ml respectively (p = 0.15, two-sample t-test). The concentration of OT in the incubation media with DRGs was 70.59 ± 7.8 pg/ml and 70.43 ± 8.6 pg/ml (p = 0.9, two-sample t-test), in females and males respectively. Incubation with TeNT significantly reduced levels of both AVP and OT in the media (Fig. 6b). The concentration of AVP, was 16.21 ± 1.15 pg/ml under control conditions and 10.02 ± 4.13 pg/ml in the presence of the toxin (p = 0.0037, n = 9). Likewise concentration of OT decreased from 48.68 ± 2.9 pg/ml in control, to 23.25 ± 6.4 pg/ml in the presence of TeNT (p = 0.0036, n = 7).

### Effects of dehydration and lactation on AVP and OT in DRG neurones: Expression of oxytocin increases during lactation

**Figure 7:**
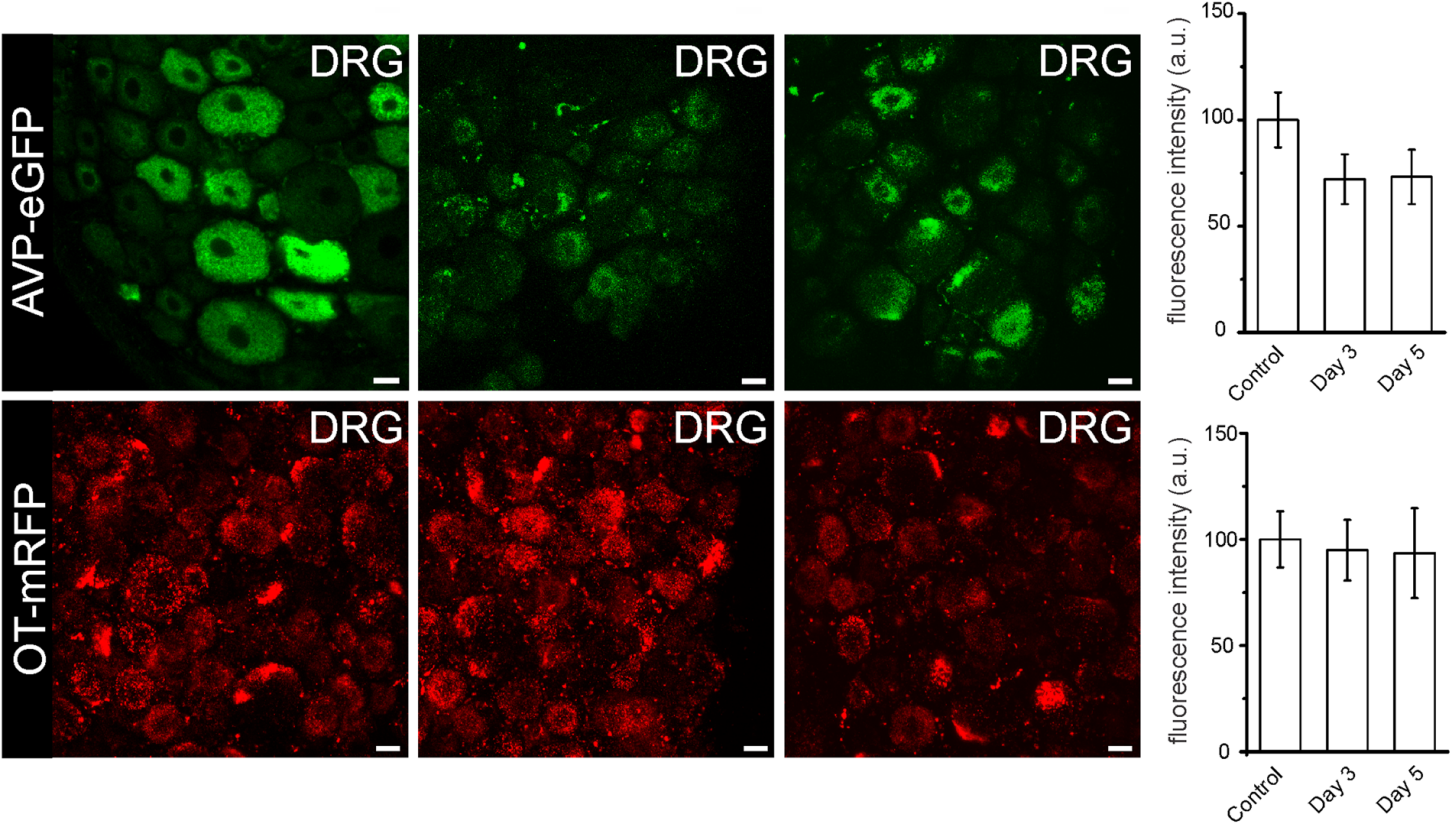
Effects of dehydration on AVP and OT expression in DRG Expression of endogenous fluorescence in DRG explants (lower panels) isolated from AVP-eGFP and OT-mRFP transgenic rats dehydrated for 3 (middle panels) and 5 days (right panels). The relative fluorescence expression in control preparations was taken as 100%. Dehydration affected fluorescence of neither AVP-eGFP nor OT-mRFP DRG explants, as quantified on bar graphs on the right. Scale bars 20 μm.

Dehydration is known to affect AVP release and expression in the neurohypophysis^21^. By monitoring specific fluorescence associated with AVP and OT we assessed expression of both hormones in DRG in three groups of animals (with three rats per group) deprived for water for 0 days (control), for 3 days and for 5 days. Expression of AVP and OT in the DRG neurones was not affected in dehydrated rats (Fig. 7).

**Figure 8:**
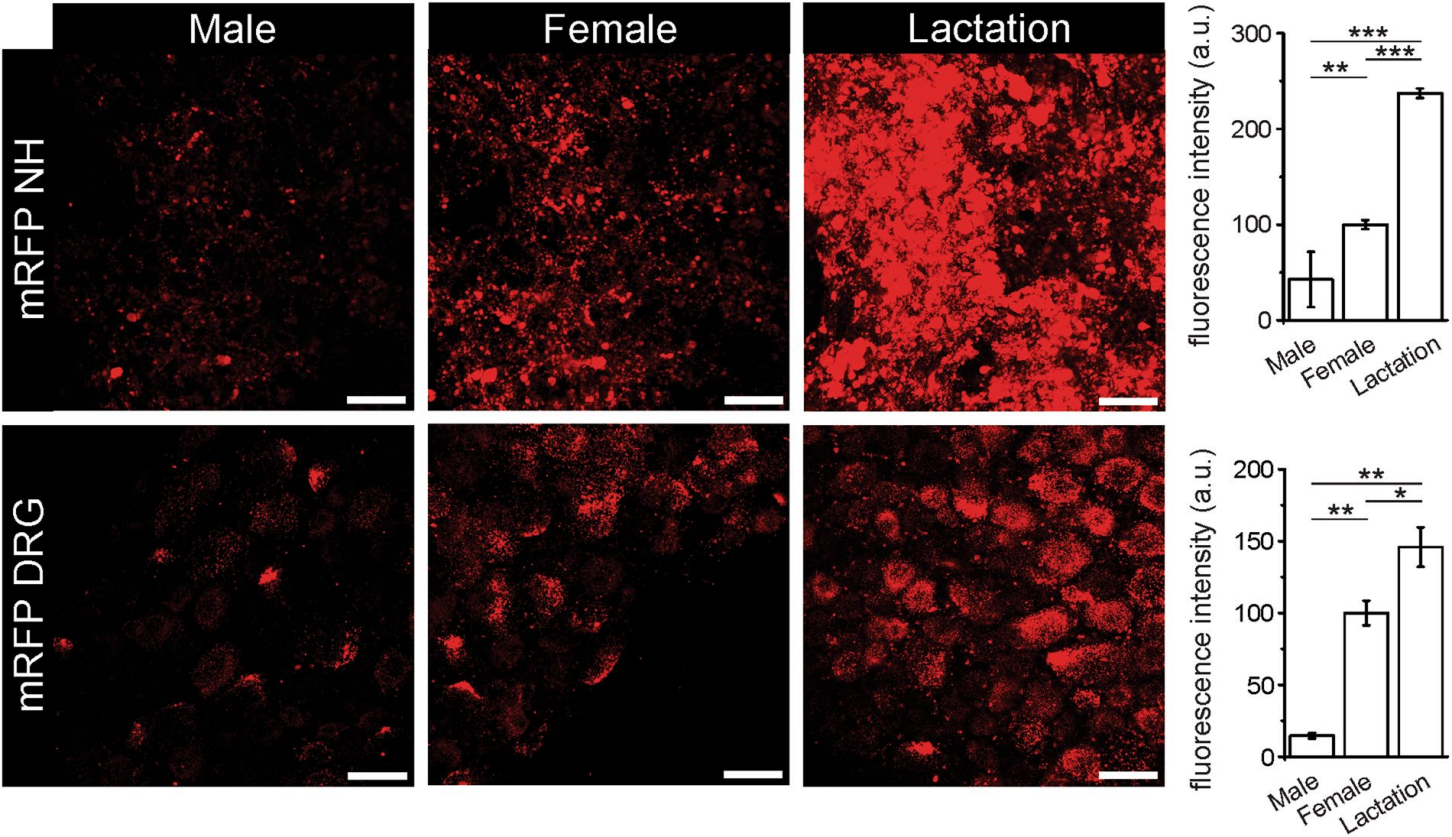
Effects of lactation on AVP and OT expression in NH and DRG. Confocal images showing the OT-mRFP fluorescence in NH and DRG of male (left panel), female (middle panel) and lactating female (right panel) transgenic rats. Expression of OT-mRFP both in NH and DRG is sex-dependent being significantly higher in females (NH, 100 ± 4.7 a.u. n = 5 p = 0.005; DRG, 100 ± 8.5 a.u. n = 4 p = 0.0003) compared to males (NH, 42.7 ± 28.9 a.u. n = 3; DRG, 14.6 ± 1.9 a.u. n = 3). During lactation expression of OT-mRFP increases more than 2 fold in NH (237.3 ± 5 a.u., p = 0.00004, n = 4) and almost 1.5 fold in DRG (145.9 ± 13.7 a.u., p = 0.0017, n = 4) compared to non-lactating females (two-sample t-test). Scale bar 50 μm.

Dynamic changes of OT expression and secretion in SON play the major role in regulation of lactation^5,30^. We monitored OT expression in NH and sensory neurones in lactating rats using OT-mRFP transgenic animals. The experimental groups consisted of four lactating female, four virgin female and three male OT-Tg rats. Expression of OT is gender-dependent being higher in females and it significantly increases during lactation in both NH and DRG. Figure 8 shows the differences in the OT expression in freshly isolated neurohypophysis and DRG explants from male (left panel), virgin female (middle panel) and lactating female (right panel) of OT-mRFP-transgenic rats indicating robust increase in OT expression in both NH and DRGs during lactation.

## Discussion

The question of peripheral expression of neurohypophyseal hormones vasopressin and oxytocin was a matter of several sporadic and controversial observations. Expression of OT and AVP in sensory neurones was reported^6–8^, but not confirmed^9–11^. In this study we used transgenic rats carrying fluorescently labelled neurohormones (AVP-eGFP, OT-eCFP, OT-mRFP1 or AVP-eGFP and OT-mRFP1); these animal models have been developed to monitor the kinetics, turnover and release of AVP and OT in neurohypophysis and other neuronal structures^19,21–23^. By employing this transgenic technology we were able to visualise DRG neurones expressing florescent protein tagged neurohypophyseal hormones AVP and OT. This expression was further confirmed by DRG staining with specific antibodies against AVP and OT. A subpopulation of AVP and OT expressing DRG neurones in addition possesses TRPV1 channels; these neurones are also sensitive to TRPV1 agonist capsaicin. These observations indicate that neurohypophyseal hormones are expressed in nociceptive neurones. In contrast to the neurohypophysis, where expression of AVP and OT is clearly segregated between separate cell populations, sensory neurones express both hormones. These hormones are secreted from sensory neurones, and this release is sensitive to the tetanus toxin that binds to and cleaves the vesicle associated membrane protein (VAMP) thus blocking exocytosis^31^. In contrast to the neurohypophysis, expression of AVP in sensory neurones is not affected by dehydration; at the same time, similarly to the neurohypophysis expression of OT in neurones is up-regulated during lactation.

Peripheral administration of neurohypophyseal hormones is known to have significant analgesic effect. For example, subcutaneous injections of AVP (at 10 μg/50 μL) to rat paw lead to a string of anti-nociceptive effects mainly mediated through V_1a_ and OT receptors. In particular the AVP-dependent analgesia was associated with reduced activity of dorsal horn neurones receiving Aδ and C-fibres, whereas activity of neurones receiving Aβ fibres remained intact^32^. Similarly, intrathecal injection of AVP reduced formalin-induced nociception; this effect was absent in V_1a_^−/−^ mice and was potentially mediated through an increase of GABA_A_ receptor mediated currents in sensory DRG neurones^33^. Administration of AVP was shown to reduce capsaicine-induced pain, which action was absent in V_1a_^−/−^ animals^34^. Both AVP and OT have been found to alleviate acidosis-evoked pain^33^. The analgesic effects of OT are mainly mediated by V_1a_ receptors, as anti-nociceptive potency of OT remains unchanged in OTR^−/−^ mice, but disappeared in animals with genetic deletion of V_1a_ receptors^16^.

The anti-nociceptive effects of AVP and OT are, most likely, associated with their ability to modulate neuronal excitability. Exposure of cultured DRG neurones to oxytocin led to hyperpolarisation, which was mediated by Ca^2+^ and NO signalling as well as by ATP-dependent K^+^ channels^35^. Oxytocin acting through V_1a_ receptors was also reported to decrease the excitability of trigeminal ganglion neurones by modulating voltage-gated K^+^ channels^36^. In addition, oxytocin reduces excitability of nociceptive neurones through specific inhibition of P2X-mediated currents^37^. Oxytocin also inhibits excitatory proton-activated ASIC channels. This inhibition follows OT-dependent activation of V_1a_ receptors with EC_50_ ~ 1 mM, with subsequent activation of calcineurin and ASICs dephosphorylation^33^.

Specific oxytocin receptors have been identified in nociceptive DRG neurones associated with C-fibres^38^. Activation of OT receptors in DRG neurones triggers protein-kinase-C mediated Ca^2+^ signalling^39^. At the same time OT was found to significantly inhibit depolarisation-induced Ca^2+^ signals in capsaicin-sensitive (i.e. nociceptive) cultured DRG neurones, probably by decreasing their excitability and voltage-gated Ca^2+^ influx^40^. Generally our experiments confirmed previous data by demonstrating that most of cultured DRG neurones responsive to capsaicin (i.e. presumed nociceptive neurones) generated Ca^2+^ signals when challenged with AVP or OT. These [Ca^2+^]_i_ transients were blocked by specific agonist of AVP V_1_ receptors and specific antagonist of OT receptors respectively, indicating expression of both receptors types. We also found that AVP/OT expressing cells responded with Ca^2+^ signals to both AVP and OT, suggesting co-expression of respective receptors subtypes in the same sensory neurone. The actual mechanism of AVP/OT anti-nociception may be multifaceted. Calcium signals induced by AVP and OT can possibly activate Ca^2+^-dependent K^+^ conductance thus decreasing neuronal excitability^41^. Alternatively, AVP/OT receptors can reduce neuronal excitability by direct action of ion channels; for example V_1a_ vasopressin receptors were shown to mediate inhibition of TRPV1 channels and stimulation of K^+^^42^.

Our findings of expression of both neurohormones in nociceptive neurones add another angle to the AVP/OT-dependent analgesia. We may hypothesise that AVP/OT expressed and released from sensory neurones provide for rapid and local analgesic effect at the level of DRGs. The release of AVP and OT is exocytotic in nature (because of its sensitivity to TeNT) and hence it is regulated by neuronal Ca^2+^ signals that may be triggered either by action potentials or by activation of ionotropic and metabotropic receptors. Of note, AVP/OT expressing nociceptive neurones also express receptors for both neurohormones, which may underlie the autocrine amplification. Expression of OT in sensory neurones is increased during lactation which may reflect a peripheral adaptive mechanism that provides for local analgesia and may add OT to circulation for regulation of systemic responses including mood and behaviour.

## Materials and Methods

### Animals

Four different transgenic animals were used in this study: (1) transgenic rats expressing arginine vasopressin fused with enhanced green fluorescent protein (AVP-eGFP)^19^; (2) transgenic rats expressing an oxytocin fused with enhanced cyan fluorescent protein (OT-eCFP)^27^; (3) transgenic rats harbouring oxytocin fused with monomeric red fluorescent protein 1 (OT-mRFP)^26^ and (4) double transgenic animals expressing AVP-eGFP and OT-mRFP to visualize both AVP and OT in the same preparation^20,43–45^. Homozygous transgenic and wild-type Wistar rats were bred and housed under normal laboratory conditions (12:12 h light/dark cycle, lights on 07:00–19:00 h) with food and drinking water available *ad libitum*. Transgenic rats were screened by polymerase chain reaction analysis of genomic DNA extracted by ear or tail biopsies before breeding and use in the experiments. All experiments were performed in accordance with the European Communities Council Directive of 24 November 1986 (86/609/EEC) regarding the use of animals in research, experimental protocols were approved by the Ethics Committee of the Institute of Experimental Medicine, Academy of Sciences of the Czech Republic, Prague, Czech Republic (project experiment license #CZ 205/2010 revised in 2013), and University of Occupational and Environmental Health, Kitakyushu-Japan.

### Experimental procedures

Between 4 and 16 weeks old, either transgenic or non-transgenic (control), male, female, and lactating (3–6 days of lactation) Wistar or transgenic rats were used in this study. Each animal was killed by decapitation after deep anaesthesia with 5% isofluran for 5 min, the brain was rapidly removed, neurohypophysis was dissected out and pars intermedia were removed within 2 min after decapitation. The neurohypophysis was then immediately observed under fluorescent microscope (AxioObserver.D1, Zeiss, Jena, Germany) equipped with eGFP, eCFP and Texas Red filters or under a Zeiss LSM 5 DUO confocal microscope (Zeiss, Jana, Germany) for green or cyan or red fluorescence. Subsequent DRG isolation was carried out only from the animals whose neurohypophyses were positive to AVP-eGFP or OT-eCFP, or OT-mRFP.

### Quantitative evaluation of endogenous AVP/OT expression *in situ*

For confocal imaging freshly isolated tissue was used. Fluorescence images of neurones expressing AVP-eGFP, OT-eCFP or OT-mRFP were taken using Zeiss Axio Observer microscope with a 40x objective and Axio Vision4 software (Carl Zeiss Vision GmbH, Germany). Three images (200 µm^2^) from each region of interest (ROI) were randomly recorded from NH and 2 DRGs from each animal. Then, the optical densities (grey scale levels of the corresponding pixels of the pre-processed image) along with the surface area were determined by means of ImageJ software. The background optical density, measured from NHs and DRGs preparations from non-Tg age-, and sex-matched controls were subtracted. All fluorescence intensities were than normalised as followed: in dehydration experiments to the control group (dehydration day 0), in lactation experiments to the non-lactating females. The experimental group for lactation experiment consisted of 4 lactating female, 4 female and 3 male OT-Tg rats. In dehydration experiment animals (three rats were used in each group) were deprived from water for 0 h, 72 h and 120 h. Dry food was always available throughout the period of water deprivation. All numbers are presented as arbitrary units. The values reported are the normalized group means of the average intensity density.

### Isolation and culture of DRG neurones

DRG neurones were isolated using procedures reported previously^46^ with minor modifications. Briefly, the rats were sacrificed after deep anaesthesia. Under a stereoscopic microscope ganglia together with 1–1.5 cm of spinal nerves were dissected from the entire length of the vertebral column after previous removing of the spinal cord. The ganglia were incubated at 37 °C in 0.2% w/v solution of collagenase type IV (Gibco) in HBSS for 90 min followed by a combination with trypsin (0.1% w/v, Invitrogen, Carlsbad, CA, USA) for another 10 minutes and then the tissue suspension was gently triturated with polished Pasteur pipette. This tissue suspension was then transferred to DMEM (Gibco) containing 10% FCS and centrifuged at 200 × g for 2 min. The supernatant containing enzymes was removed gently. The pellet containing cells was resuspended in 10% FCS solution and carefully layered on 15% BSA in DMEM. After centrifugation at 120 × g for 15 min, the supernatant was removed and the pellet was suspended in DMEM containing 10% FCS and centrifuged at 200 × g for 2 min. Finally, the pellet which contained cells were resuspended in culture medium containing DMEM, 2% B27 (Gibco), 100 U/ml of penicillin (Invitrogen), 100 ng/ml of streptomycin (Invitrogen), 100 µl mitomycin C (MMC, Sigma), and NGF 25 ng/ml (Sigma) and cultured on cover slips and on 24 mm glass-bottom dishes (WillCo-Wells Dishes-BV, Amsterdam, Netherlands) coated with Laminin (Sigma). The cells were kept at 37 °C in a humidified atmosphere of 95% air and 5% CO_2_.

### Solutions and drugs application

The Normal Locke’s (NL) buffer was used during dissection and as a control solution. It contained (mM): NaCl, 140; KCl, 5; MgCl_2_, 1.2; CaCl_2_, 2.2; glucose, 10; HEPES-Tris, 10, BSA, 0.02%, pH 7.25. The osmolarity of all the solutions used in this study was maintained at 298–300 mosmol/l-1. Buffer containing high K^+^ contained (mM): NaCl, 90; KCl, 50; MgCl_2_, 1.2; CaCl_2_, 2.2; glucose, 10; HEPES, 10 at pH 7.25. For other K^+^ concentrations, KCl was added at the desired concentration and was adjusted with NaCl appropriately to bring the osmolarity to the required range. AVP, OT capsaicin and capsazepine were purchased from Sigma. Concentrated stock solutions of capsaicin and capsazepine were prepared in DMSO and further diluted in working solution to appropriate concentrations (1 μM and 10 μM respectively). Stock solutions of AVP and OT were prepared in distilled water and then diluted to working concentrations in the NL buffer before use. In physiological experiments the control and test solutions were applied using a temperature controlled multichannel polypropylene capillary perfusion system (Warner Instruments, Inc., USA). A single outlet capillary tubing (100 μm inner diameter) with a flow rate of 250 µl/min was positioned close to the tested cell (<0.5 mm). Selected cell was subjected to a constant flow of control buffer or test solutions. Each capillary was fed by a reservoir 45 cm above the bath and connected to a temperature control device (Harvard-France). The temperature of all solutions was maintained at 37 °C. In this approach, switching the flow from one capillary to the next resulted in complete solution exchange within 1–3 seconds. After each application of the tested drug, the cells were washed with control buffer. This method allowed for the fast and reliable exchange of the solution surrounding the selected cell under observation without exposing the neighbouring cells.

### Single cell [Ca^2+^]_i_ recordings

Cultured DRG neurones were incubated with 2.5 μM Fura-2 AM supplemented with 0.02% Pluronic F-127 at 37 °C for 40 min; subsequently cells were washed and the culture medium replaced with Normal Locke’s buffer and kept at 37 °C throughout the experiment. The details of [Ca^2+^]_i_ measurements on single cells using fast fluorescence microspectrofluorimetry have been described previously^47^. Fura-2 calibration was performed following the procedure described elsewhere^47–49^ and yielded R_min_ = 0.225, R_max_ = 3.816, β = 3.437 and K_d_ of 224 nM at 37 °C. The CCD-based Ca^2+^ imaging experiments were performed as described in^43^. The calibration performed with the imaging system gave R_min_ = 0.2, R_max_ = 7.2, β = 7.7, with Fura-2 K_d_ 224 mM. Experiments were performed on 6 different cultures prepared from AVP-eGFP rats and 6 different cultures prepared from OT-mRFP rats. From each culture 3–5 coverslips were used. Prior to the experiment AVP/OT expressing neurones were identified using eGFP or mRFP fluorescence using an appropriate filter set.

### *In vitro* immunocytochemistry

Cells plated onto laminin-coated coverslips were washed in phosphate-buffered saline (0.1 M PBS, pH 7.2) and fixed with 4% paraformaldehyde in PBS for 15 min. The fixed cells were washed twice in PBS prior to immunostaining. Permeabilization and blocking were carried out in a blocking buffer consisting of 0.4% Triton-X 100, 10% bovine serum albumin in 0.1 M PBS for 45 min at room temperature (RT; 24 °C). Primary antibodies were diluted in buffer consisting of 0.1% Triton-X 100, 2% bovine serum albumin in PBS overnight at 4 °C. After 2 washes with PBS, appropriate secondary antibodies were applied for 30 min at RT. To visualize the cell nuclei, the coverslips were incubated with 300 nM 4′, 6-diamidino-2-phenylindole (DAPI) in PBS for 5 minutes at RT. Finally, the coverslips with cells were mounted using Aqua Poly/Mount mounting medium and examined using a ZEISS LSM 510 DUO confocal microscope (Carl Zeiss, Jana, Germany).

### AVP and OT assay

We have employed Enzyme-Linked Immunosorbent Assay (ELISA) method for the detection of AVP and OT peptides in the incubation media containing whole DRGs from adult Wistar rats (4 weeks old) under normal conditions (control; Leibovitz media without phenol red (Cat.no 21083027, Gibco, Czech Republic) and in the presence of tetanus toxin (TeNT). The competitive ELISA kits for the detection of OT (ab133050) and AVP (ab205928) were purchased from Abcam, (Biotech, Czech Republic). DRG were dissected from cervical, thoracic and lumbar levels of 2 male and 2 female rats, cleaned from connective tissue and placed into the 12 mm wells (10 ganglia per well) filled with: (a) 600 μl of phenol-free Leibovitz media (control group); or (b) 600 μl of phenol-free Leibovitz media with 100 nM TeNT (tetanus toxin group). All samples were incubated overnight at 37 °C with a controlled CO_2_ concentration (5%). Subsequently, incubation media from control and tetanus groups were collected for further analysis. Release was measure in triplicates for both OT and AVP ELISA assays separately from males and females. Assay buffers, serially diluted standards and samples were prepared following manufacturer’s instructions freshly before the assay procedure. The peptides concentrations were determined by reading the optical density at 405 nm (OT) and 450 nm (AVP) using a plate reader (BioTek-EL808).

### Data analysis and statistics

Origin 8.5.1 and MATLAB-MathWorks Statistics Toolbox were employed for plotting and statistical procedures. The results are expressed as mean ± SEM. The number of the sample size (n) given is the or cells tested in [Ca^2+^]_i_ measurements according to the same protocol (control, test drug, recovery) for each group. The figures (traces) show single cell recordings of the [Ca^2+^]_i_ before and after the application of test substances. Repeated measures ANOVA applied to these experiments showed that that the means were not all equal in the measured conditions (p < 10^e-6^) The Andereson-Darling and the Lilliefors tests for deviations from normality, as well as Mauchly test for deviations from shericity were negative. While this supports the validity of the ANOVA analysis, we also carried out the analogues non-parametric Friedman test, confirming the finding of non-equal means (P < 0.01). Post-hoc pairwise comparisons (t-test for Bonferroni correction) identified statistically significant differences between the antagonist and control conditions (p < 3e–4 for each parwise comparisons). No significant diference was found between the two control conditions in eisther dataset.

The results obtained from ELISA toxin studies (Fig. 6b) were analysed using one-way ANOVA. Prior to the One-way ANOVA test, the Mauchly’s test of Sphericity was performed and if the value of Prob > Chi_Sq_ was ≥0.05, the data were further used for analysis. The data obtained from immunocytochemical studies (Figs 7 and 8) and analysis of AVP/OT expression in male vs. female studies (Fig. 6a) have been analyzed using Student’s t-test. Differences were considered statistically significant at p ≤ 0.05
